# Scaling oscillatory platform frequency reveals recurrence of intermittent postural attractor states

**DOI:** 10.1038/s41598-018-29844-2

**Published:** 2018-08-01

**Authors:** Aviroop Dutt-Mazumder, Troy J. Rand, Mukul Mukherjee, Karl M. Newell

**Affiliations:** 1 Department of Physical Medicine & Rehabilitation, University of Michigan, Ann Arbor, United States; 2 Department of Biomechanics, University of Nebraska, Omaha, USA; 3 Department of Kinesiology, University of Georgia, Athens, USA

## Abstract

The study of postural control has been dominated by experiments on the maintenance of quiet upright standing balance on flat stationary support surfaces that reveal only limited modes of potential configurations of balance stability/instability. Here we examine the self-organization properties of postural coordination as revealed in a dynamic balance task with a moving platform. We scaled a control parameter (platform frequency) to investigate the evolving nature of the coupled oscillator dynamics between center of mass (CoM) and platform. Recurrent map measures were used to reveal whether episodic postural control strategies exist that can be scaled by systematically changing the magnitude of platform motion. The findings showed that at higher platform frequencies (1.2 Hz), the CoM-Platform coupling was less deterministic than lower platform frequencies and evolved to intermittent postural control strategies that oscillated between periodic-chaotic transitions to maintain upright postural balance. Collectively, the recurrence map measures indicated that quasi-static postural attractor states were progressively emerging to the changing task constraints of platform frequency in the maintenance of postural stability. It appears that several dynamic modes of intermittent coupling in postural control can interchangeably co-exist and are expressed as a function of the control parameter of platform frequency.

## Introduction

Postural control encompasses non-linear interactions of subsystems involving sensory and motor processes that collectively organize to maintain postural balance^1,2^. The maintenance of equilibrium requires integration of these sensorimotor strategies to stabilize the body’s center of mass (CoM) during both internally and externally triggered perturbations^3^. A critical component of postural control is the ability to self-organize the motion of the torso and limb segments with respect to the gravity, support surface and sensorimotor information cues. Perturbations of quiet standing provide broader insights into the self-organization properties of postural coordination by moving the system through an enhanced range of postural states that could reveal strategies how to avoid a fall^4^.

The emergent nature of postural coordination is an expression of self-organization in biological systems. The self-organization leads to adaptive synergies that are task dependent across the postural degrees of freedom (*dof*), defined over multiple scales of space and time^5,6^. The adaptive functioning of the postural system can be interpreted as discovering a low dimensional collective variable that encapsulates the underlying spatial-temporal patterns among the *dof* within the movement system^7^. The construct holds parallels to the role of macroscopic variables in other systems’ frameworks of essential variables^8^ and order parameters^9,10^ that are embedded in many disciplines ranging from the physical to biological sciences^11,12^. An open question in motor control and, that we investigate here, is whether the scaling of a control parameter (externally triggered perturbation of oscillating platform frequency) would reveal any intermittent postural control strategies (quasi-static postural attractor states). In this context of postural stability, intermittent modes of coupling in postural control reflect the transient states between periodic deterministic and aperiodic stochastic processes^13,14^.

Many studies have investigated the coordination and stability of the postural system during the act of quiet upright standing^15,16^. There have also been studies of postural control in a discrete perturbation dynamic postural balance task^17,18^, supra-postural bimanual task^19^ and continuous oscillatory platform motion^20–22^. In both anterior-posterior^23^ and medio-lateral^24^ moving platform postural tasks, CoM motion to that of platform motion remained in-phase or transitioned to anti-phase as a function of the platform frequency. These results have provided preliminary evidence that the relative coupling of center of mass (CoM - an emergent macroscopic property) with respect to the oscillatory platform motion could be considered as a collective variable for upright postural stance task^23,25^.

A postulation of coordination dynamics is that by scaling a control parameter (e.g., platform frequency), it is possible to discern the intermittent postural control strategies while maintaining the same upright balance task. Theoretically, the reciprocal control between the slower time scales of the emergent collective variable and the faster time scales of the local synergies^26–29^ in the regulation of movement and posture should indicate these episodic postural control patterns^3,30^. This distinction between the role of the variables (joint synergies and collective variable) is fundamental to understanding the multiple *dof*s case of postural control. Previous studies on oscillatory platform motion over various time scales have demonstrated the convergence towards and divergence from stable postural coordination between CoM and platform motion^22,24^. Other studies have also illustrated the chaotic dynamics^31,32^ and fractal properties of postural swaying during upright quiet standing^33,34^, and also in human locomotion^35^. However, progressive scaling of platform frequency would provide us a closer insight into the postulated intermittent coupling of CoM-platform oscillators, their nature of emerging attractor states, and the duration of trapped attractor states.

One-way to examine the intermittent coupling of CoM-platform oscillators is with recurrent maps^36^, that can reveal dynamic properties that are unavailable in relative phase analysis^37^. The method affords the capture of high-dimensional interactions between CoM-platform phase trajectories, that otherwise would be very difficult to visualize. Structural patterns in recurrent maps can reveal hints about the time evolution of these phasic trajectories^38^. Another distinct advantage of recurrent maps is that they exhibit both large-scale and small-scale patterns. A closer inspection of such maps identifies a combination of *isolated dots* within small-scale structures (chance recurrences), dots forming *diagonal lines* (deterministic structures), as well as *vertical/horizontal lines* or dots clustering to inscribe *rectangular laminar states* (singularities). The length of the *diagonal lines* is directly related to the ratio of determinism to the dynamics. In other words, a perfectly predictable system would have infinitely longer *diagonal lines* in recurrent maps, whereas a chaotic system will depict single *dots* or shorter *diagonal lines*. The *vertical lines* reflect a time length in which a state does not change or changes very slowly (trapped states). This is a typical behavior of *laminar* states (intermittency) or systems that paused at singularities. These small scale structures are the basis for quantitative analysis of the recurrence plots^39^.

In this investigation we applied cross recurrence quantification analysis (cRQA), that is a bivariate extension of recurrence plots and was introduced for the investigation of the simultaneous evolution of two different phase space trajectories (e.g. CoM and platform motion)^40,41^. An important advantage of cRQA is that it allows the study of evolving dependencies between two systems^42,43^. We examined the hypothesis that by increasing platform frequency the postural system will shift from a more tightly coupled control strategy to an intermittent coupling strategy^44^. Tighter coupling would be reflected by longer *diagonal lines*, i.e. highly deterministic at lower platform frequencies. Scaling up of platform frequencies, it is hypothesized will loosen the coupled system, i.e. shorter *diagonal lines*, and simultaneously reveal intermittent postural control strategies, i.e. longer *vertical lines*.

We predicted that the CoM-Platform coupling would be highly non-stationary with shorter quasi-periodic balance strategies at the high platform frequencies as reflected by lower *%determinism*. We used *%laminarity* for recurrence points in vertical lines that is analogous to % determinism for recurrence points in diagonal lines. This measure is directly related to the number of laminar phases in the system (intermittency). We also anticipated that as the CoM-Platform coupling evolved the Euclidean radius should differentially increase from lower platform frequencies to higher platform frequencies. By comparing these cRQA measures across a range of increasing and decreasing platform frequencies we can determine if any intermittent postural mechanism is evolving as a function of the control parameter. The more standard measures of postural control on this moving platform data set may be found in^24^.

## Results

**Figure 1:**
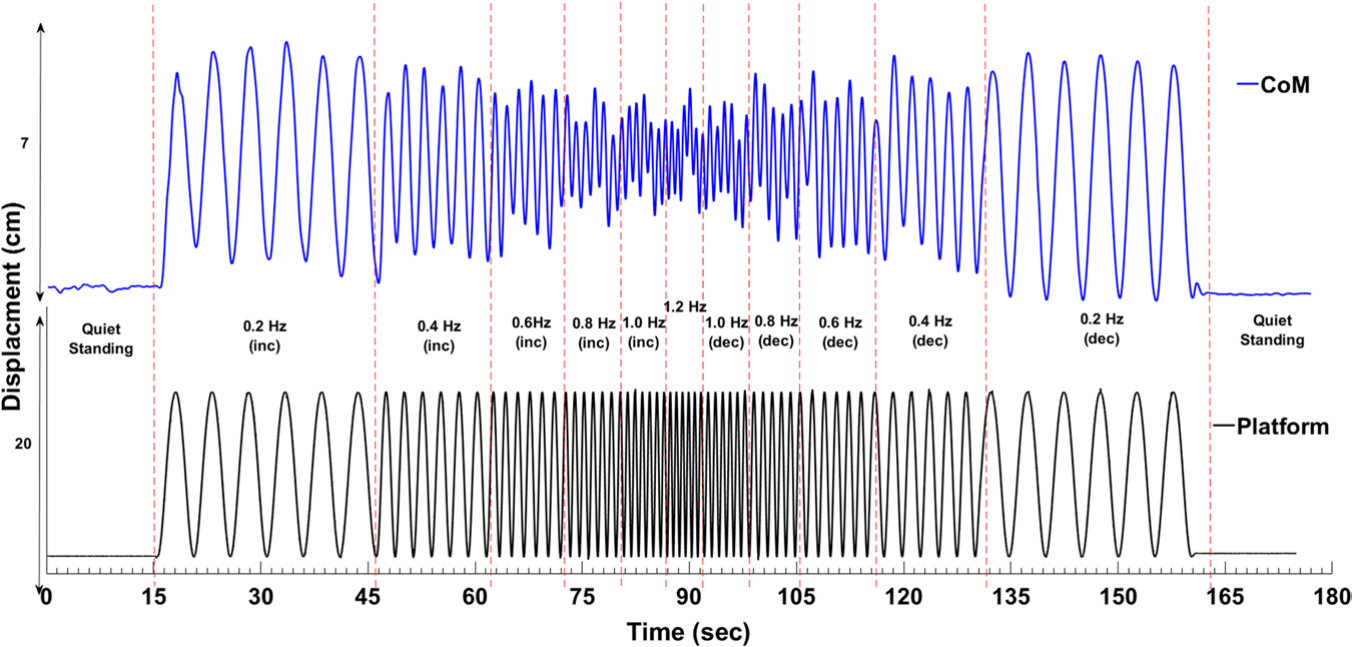
Representative medio-lateral center of mass (CoM), and Platform motion time-series measured during scaling of the platform frequency, starting from quiet standing, 0.2 Hz, 0.4 Hz, 0.6 Hz, 0.8 Hz, 1.0 Hz, 1.2 Hz and scaled back to quiet standing. Each platform frequency lasted for six complete cycles. Cross recurrent map measures were performed on these two time-series to find dynamic similarities in their behavior.

In brief, CoM and platform motion were determined using 3-D motion capture and a force platform with appropriate standard methodology (see methods section). The participants were instructed to maintain their upright postural balance while the platform oscillated sinusoidally in the ML plane over a range of platform frequencies. The CoP-platform coupling was analyzed using cross recurrence measures to reveal their underlying attractor dynamics as a function of platform frequency (see Fig. 1). We employed one-way repeated measures of ANOVA to test the effect of frequency on mean line length and *entropy* values for *diagonal* and *vertical* lines, *radius*, %*determinism*, and %*laminarity*. The central rationale of this investigation was primarily based on the idea that by scaling a control parameter (e.g., platform frequency), it is possible to discern the intermittent postural control strategies using the cRQA variables.

**Figure 2:**
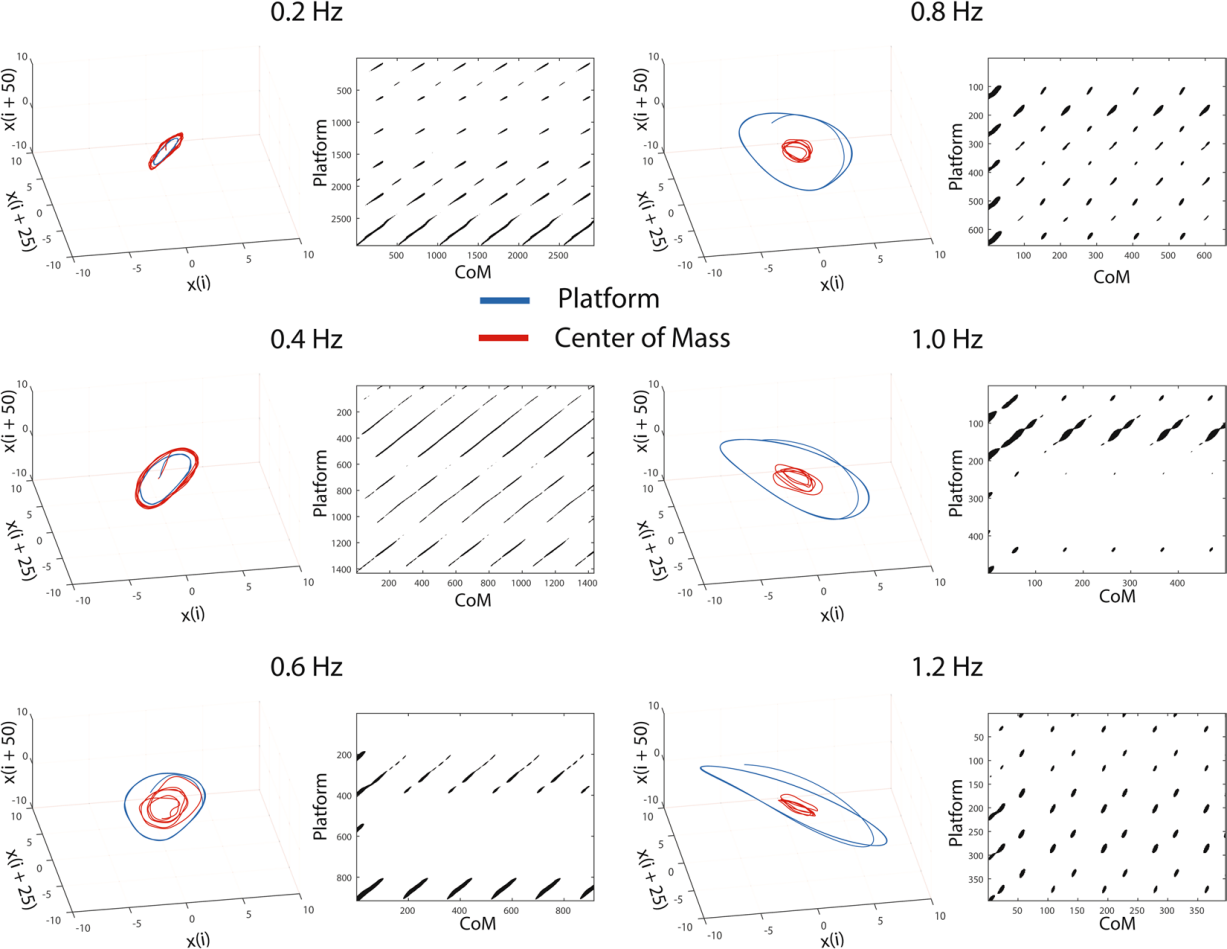
Example attractors and recurrence plots for one representative participant at two different frequencies. The 3-dimensional attractor from 0.2 Hz platform frequency to 1.2 Hz platform frequency. The CoM-platform transgress from a tighter coupling to an intermittent control strategy.

Figure 2 shows representative recurrence plots of platform frequencies from 0.2 Hz to 1.2 Hz with tighter coupled CoM-platform oscillators at 0.2 Hz (longer *diagonal lines*), to an intermittent coupled oscillator at 1.2 Hz (shorter *diagonal lines*).

### Control Parameter Sensitivity to Postural Robustness

**Figure 3:**
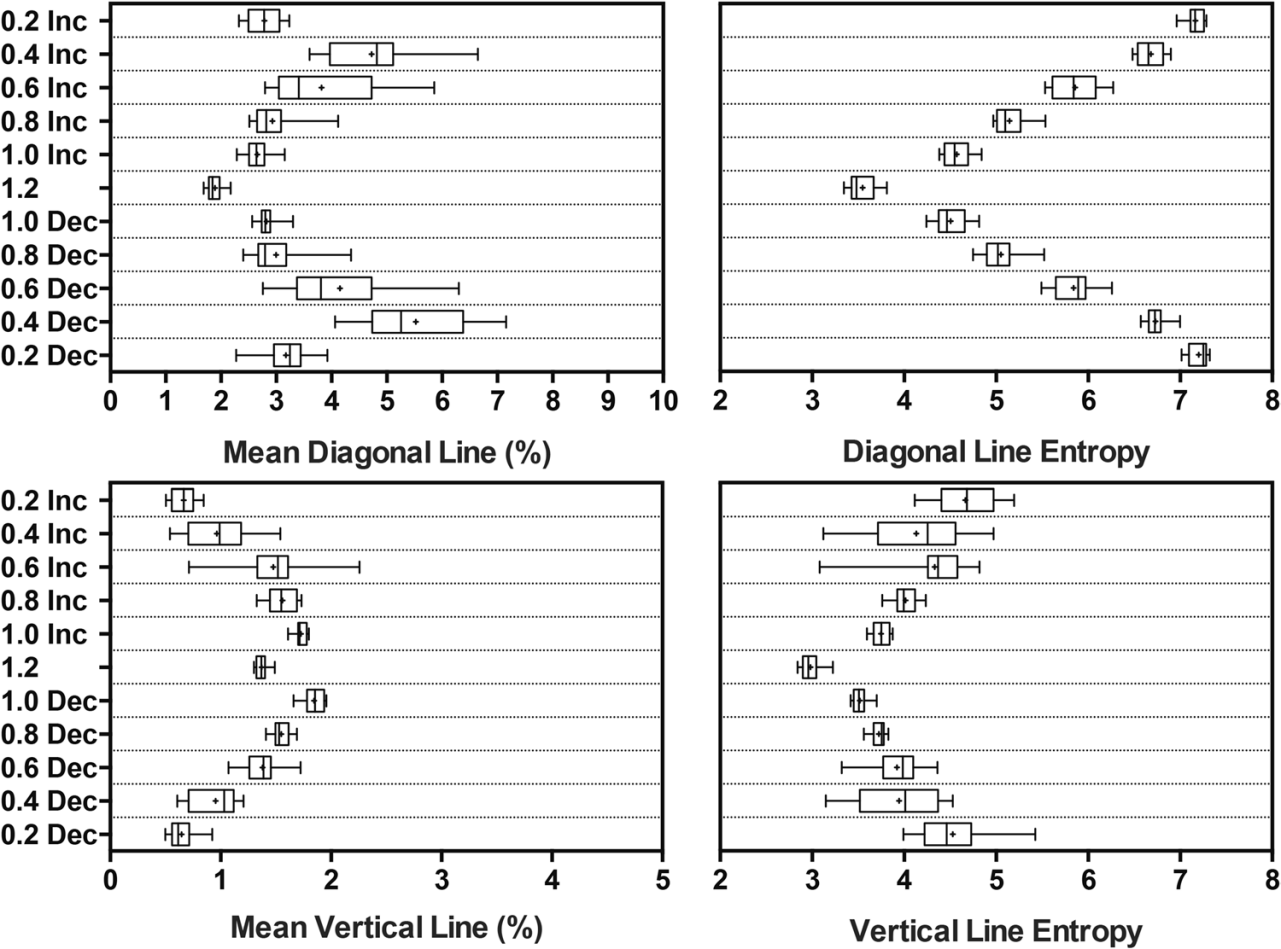
Box and whisker plots of cRQA variables showing the data range from minimum to maximum (error bars), 25^th^ and 75^th^ percentiles (box edges), median value (straight line), and mean value (plus sign). Mean *diagonal line* (top left) shows that initially there is an increase in the coupling and then a decrease as the frequency increases. The entropy of *diagonal line* lengths (top right) decreases as platform frequency increases. Mean *vertical line* (bottom left) increases as platform frequency increases except it decreases for the highest frequency. Entropy of *vertical line* lengths (bottom right) decreases as platform frequency increases. Note that the x-axis scale on *vertical mean line* is half of the *diagonal mean line*.

The results for *vertical* and *diagonal* mean line and *entropy* of line lengths are displayed in Fig. 3. There was a significant effect of platform frequency on cRQA mean *diagonal line* and *diagonal line* entropy as determined by one-way repeated measures ANOVAs (*F*(10, 130) = 47.86, *p* < 0.0001; *F*(10, 130) = 908.5, *p* < 0.0001, respectively). In this context longer cRQA mean *diagonal line* reflects the stronger synchrony between CoM and platform motion. Likewise, tightly synchronous oscillators consisting of CoM and platform motions should have longer entropy *diagonal lines. Entropy* of line length decreased as frequency increased and then increased as frequency decreased. Tukey post-hoc comparison revealed that each frequency was different from all other frequencies at *p* < 0.0001 level. The boxplots indicate that the variability of *entropy* of line lengths was highest between platform frequencies 0.4 and 0.6 Hz. Also, there was no difference in the increasing and decreasing values at a given frequency (e.g., 0.2 Hz increasing vs. 0.2 Hz decreasing), except for 1.0 Hz increasing and decreasing (*p* = 0.024). The post-hoc also showed a general trend of mean line was an increase from 0.2 to 0.4 Hz, then a decrease from 0.4 to 1.2 Hz, as frequency decreased the mean line increased from 1.2 to 0.4 Hz and then decreased from 0.4 to 0.2 Hz.

### Higher Entropy and Deterministic Postural Processes at Lower Platform Oscillations

There was a significant effect of platform frequency on cRQA mean *vertical line* and *vertical line* entropy as determined by a one-way repeated measures ANOVAs (*F*(10, 130) = 104.9, *p* < 0.0001; *F*(10, 130) = 39.5, *p* < 0.0001, respectively). Unlike the *diagonal lines*, the *vertical line* lengths increased with increasing platform frequencies, until the highest frequency where there was a decrease. Post-hoc analysis revealed that the entropy of *vertical lines* followed a similar pattern to the entropy of *diagonal lines*, decreasing with increasing platform frequency.

### Intermittent Control Strategy Expressed at Higher Platform Frequencies

**Figure 4:**
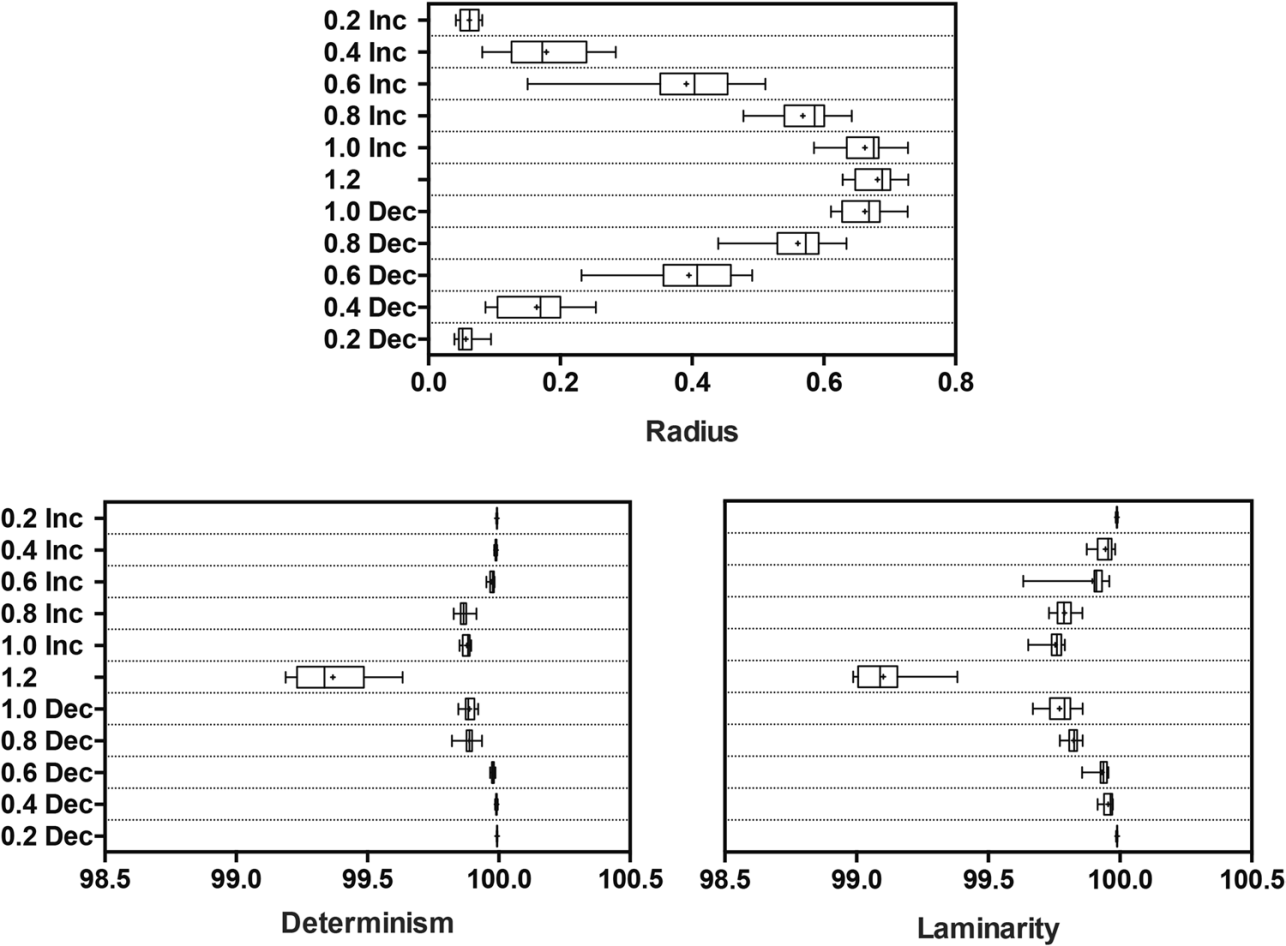
Box and whisker plots using the same convention as Fig. 4. The radius (top) increased as platform frequency increased. Both %*determinism* (bottom left) and %*laminarity* (bottom right) were almost 100% for all conditions, but there was a significant decrease at the highest frequency.

The results for radius, *%determinism*, and *%laminarity* are displayed in Fig. 4. There was a significant effect of platform frequency for radius (*F*(10, 130) = 536.0, *p* < 0.0001), *%determinism* (*F*(10, 130) = 221.5, *p* < 0.0001), and *%laminarity* (F(10, 130) = 392.7, *p* < 0.0001). In the context of postural coordination, systems possessing deterministic dynamics are characterized by longer *diagonal lines*, and hence have a more predictable periodic behavior than a chaotic process. Concurrently, *laminarity* reflects the frequency of laminar states (intermittency) with the CoM and platform oscillators. *Radius* increased with increasing platform frequencies, except for no change between the two highest frequencies. The %*determinism* and %*laminarity* were almost 100% for all conditions. However, there were statistically significant reductions in both variables as the frequency increased, and significant increases as the frequency decreased.

## Discussion

Employing a strictly operational approach in which theory and experiment go hand in hand, we have provided empirical evidence that there are quasi-static attractor states, reflecting differential online postural control strategies as revealed through scaling of the platform frequency (control parameter). The novelty of this study is that by scaling platform frequency, the CoM-platform oscillator transgresses from a very deterministic system to periodic-chaotic intermittent postural states. Such categorical distinction of attractor dynamic states within platform frequencies has not been explicitly captured in a quiet standing task. This is primarily because postural stability on an oscillating platform requires resisting external triggered perturbations, unlike resisting natural internal perturbations during a quiet standing task^3,45^. By investigating these recurrence maps, cRQA revealed important features that shed light on the intermittent postural control mechanisms and processes.

First, we observed a significant effect of platform frequency on the mean *diagonal* and *vertical* lines for the CoM-platform oscillators (see Fig. 3). Furthermore, there was an inverse relationship between the *diagonal* and *vertical* lines. These line structures contain the information about epochs of a similar evolution of phase space trajectories^46^. Shorter mean *diagonal lines* along with longer *vertical lines* at higher frequency indicate that the coupled motions are moving together for shorter periods of time but employing slow dynamics or intermittent coupling for a greater period. Also, at 1.2 Hz the distribution of diagonal lines resemble *isolated dots* and varying short lengths that are typical of a chaotic system^47^.

Hence, from this perspective, the dynamics of CoM-platform motion at 1.2 Hz are less deterministic than lower platform frequencies. This supports the view that several dynamic modes of intermittent postural control strategies can interchangeably co-exist and that are scalable via deterministic perturbations of platform frequency^30,48^.

Second, entropy lowered considerably for both *diagonal* and *vertical* lines as a function of higher platform frequencies, which reflects that CoM-platform dynamics have lower complexity for ≥0.6 Hz (see Fig. 2). This result complements earlier investigations which have shown that for CoM and CoP coupling, there was a phase transition between 0.4 Hz and 0.5 Hz (anteroposterior)^23^, and between 0.4 Hz and 0.6 Hz (mediolateral)^24^. A typical feature of phase transition, reflects higher variability^49^, and our results indicate that the variability of entropy was highest between platform frequencies of 0.4 Hz and 0.6 Hz. Also, as predicted a tighter coupled oscillator should have longer entropy *diagonal lines*, and likewise periodic-chaotic states should reveal shorter entropy *vertical lines*. This is comparable to a mechanistic simplification where high platform frequencies require a simpler postural control strategy such as a two-segmented inverted pendulum to maintain upright balance^50^.

This brings us to the next intriguing finding, where we capture large variability within the *entropy* and line lengths for both directions (*diagonal* and *vertical*) between 0.4 and 0.6 Hz. Based on our previous findings^22,24^, we found that the CoM-platform switched from in-phase to anti-phase mode of oscillation between these platform frequencies which met the criteria of a non-equilibrium phase transition in a coordinated biological motion^37^. The higher variability in *entropy* and *line* lengths in recurrent maps shows that at these platform frequencies, the postural coordination warranted a wider range of postural stability search strategies to maintain upright balance.

We found that *%determinism* was closer to 100 across lower platform frequencies. However, *%determinism* showed a decreasing trend with increasing platform frequencies, and reduced substantially at 1.2 Hz, indicating a more stochastic and less predictable structure for CoM-platform coupled oscillators. This could be possibly due to the differential self-organization of joint synergies encompassing varying anthropometric scales across individual participants^3,24,51^. On the other hand, at lower platform frequencies, the postural control strategies are less assumptive with a steady state and reduced temporal variation^52^. This finding of differential coupling illustrates the presence of different levels of postural stability strategies and is consistent with the dynamics emerging from the interaction of the organismic, task and environment constraint interactions^53^.

Figure 2 illustrates that phase space trajectories, where CoM moves with the platform motion at 0.2 Hz and all the way up to 1.2 Hz. At 1.2 Hz, the CoM moves in a similar pattern, but the magnitude of platform motion does not allow it to synchronize well. As a consequence, the system is induced to switch to an intermittent postural control, where the CoM follows the motion lead by the platform. However, the CoM constrains itself to oscillate in a conservative ‘*comfortable*’ state, while the platform motion moves beyond this CoM’s ‘*comfortable*’ state. Thereafter, once the platform motion is scaled back, the CoM follows the platform motion again, i.e. CoM-platform is intermittently synchronizing at higher frequencies since the platform movement is larger than the ‘*comfortable*’ state of CoM motion.

Drawing on the hypothesis of Riley *et al*.^54^, such conservative CoM non-stationarity motions can be explained as an intrinsic feature of dynamic stabilization during unstable platform perturbations while maintaining an upright balance by simplifying the postural control strategies of an inverted pendulum. One such strategy in postural balance is the ‘*running strategy*’ as observed by Treffner and Kelso^55^, wherein relatively quasi-periodic displacements result in fluctuations within the postural stability time series. In the context of a moving platform, the strategy is ‘*riding the platform*’^20^, where the ‘*running strategy*’ must be countered through appropriate muscular action, rather than resisting the dynamics of the system until a constraint such as stability boundary threatens the task performance^22^.

Lastly, *%laminarity* that reflects the trapping time of the coupled oscillators is lower at the highest platform frequency of 1.2 Hz. Collectively, these recurrent map measures suggest that the underlying postural control strategy in upright standing on a moving platform is more intermittent, obviated by the relative number of laminar phases in the observed postural dynamics. Alternatively, the coupled system’s propensity for intermittency was observed in relatively shorter phases when the platform oscillated at 1.2 Hz. These results are consistent with the control mechanisms that capitalizes ‘*riding the platform*’ dynamics with minimal displacements that threaten crossing the stability boundary^20,23^.

In summary, the platform frequency manipulations revealed significant differences about how postural task demands resulted in different postural dynamics. Intermittency in the postural dynamics reflects greater relative contribution from small amplitude, random fluctuations on a faster timescale, particularly at higher platform frequencies, i.e. transient modes between periodic deterministic and aperiodic stochastic processes. In addition, the differential increments of Euclidean radius with increasing platform frequencies revealed that there were more consecutive recurrent points in the reconstructed phase space.

## Conclusion

These findings both corroborate and extend previous work on dynamic postural control and reveal four important features: (a) evidence for differential online postural stability strategies, (b) intermittent postural strategies emerge at faster platform motions, that are quasi-static and interchanges to maintain postural stability, (c) magnitude of perturbation dictates the directional drift of these quasi-static postural attractor states, and (d) markedly higher variability in *entropy* and *line* lengths at phase transition warrants a wider range of postural stability search strategies in order to maintain upright balance. The postural dynamics behavior at higher platform frequencies is not limited to their deterministic properties but likely integrates intrinsic passive mechanical properties of the limb linkages. To conclude, it appears that several dynamic modes of intermittent coupling in postural control can interchangeably co-exist and differentially emerge through systematic scaling of the control parameter i.e., platform frequency.

## Methods

### Participants

The experiments were carried out according to the experimental protocol approved by Institutional Review Board of The Pennsylvania State University. All participants gave written informed consent for study participation. Eleven healthy male participants (age: 20–28 years; mass: 65–72 kg; height: 163–181 cm) free from neurological disorders and musculoskeletal injuries were recruited for the experiment. The participants self-reported that they had no previous exposure to dynamic balancing tasks such as skiing, surfing and rollerblading.

### General Setup

Passive markers attached over the joints of the experimental participants were recorded by a 3-D motion analysis system (QTM, Sweden) to reconstruct a 13-segment model from a 20- marker system to derive the CoM according to the anthropometric model data of Dempster^56^. Applying the weighting factors of the segmental masses, the total body CoM position was estimated by the weighted summation of the individual segment CoM positions^57^. Passive kinematic markers were placed on the moving platform to capture the motion along the ML direction. The motion analysis system and the moving platform device were synchronized with the aid of an external trigger. Low noise amplifiers ensured peak signal noise ratio was not reduced. Both systems were set at 100 Hz as the sampling frequency to record data that were later filtered by a low-pass second order Butterworth filter with a cut-off frequency at 4 Hz.

**Figure 5:**
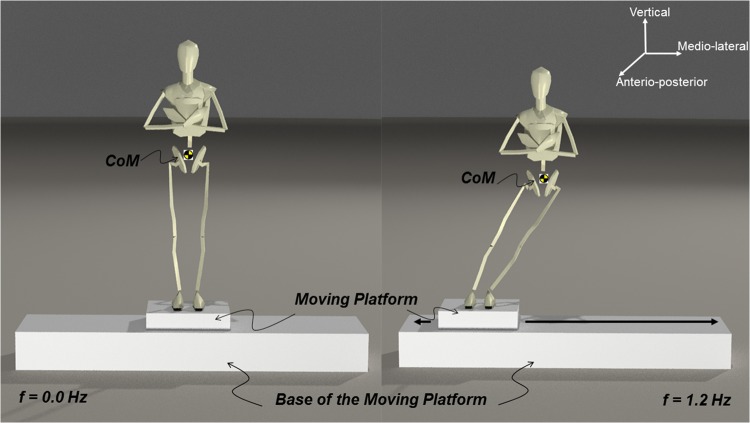
Schematic stick diagram of the experimental apparatus and setup shows the embedded force platform on a moving platform that oscillates sinusoidally along the medio-lateral direction, with a participant standing with bare feet, eyes open and arms folded. (left panel: *f* = 0.0 Hz), (right panel: *f* = 1.2 Hz).

In the experiment, participants were instructed to maintain their postural balance when they stood on a sinusoidal translating platform with bare feet eyes open and focused at a distant visual target placed 2 m away from the platform at the eye level – a layout designed to enhance motion perspective and parallax. To alleviate any optic flow, participants’ bodies were aligned parallel to the environmental arrangement (they directly faced the arrangement). The participants placed their feet side-by-side (foot width aligned with shoulder width) comfortably and kept their arms folded across their chest (see Fig. 5). An external motor that generated sinusoidal lateral translations along the ML direction controlled the moving platform. There were 6 frequencies of 0.2, 0.4, 0.6, 0.8, 1.0 and 1.2 Hz at the single platform motion amplitude of 20 cm. The initial 15 s consisted of quiet standing with no motion of the platform, followed by 6 oscillation cycles of each frequency in ascending step-wise order and then again descending order followed by another 15 s of quiet standing. The total duration of each trial was about 170 s. There were two sessions and for each session, the participant had a trial block of 6 trials, with ample recovery time between each trial to reduce fatigue. Subjects were asked to maintain their postural balance irrespective of the scaling of the platform frequency.

### Parameter selection

The CoM and platform motion along ML direction were assigned as the two time-series (see Fig. 5). Then we reconstructed these time-series to phase space trajectory using the standard time delay method^36,54^, involving an embedding dimension (*m*) and time lag (τ). The embedding dimension is the number of state space dimensions and the lag is how far the time series is offset when creating the higher dimensions. The preservation of topological structures of the original trajectory is guaranteed if *m* ≥ 2*d* + 1, where *d* is the attractor dimension^58^. We used false nearest neighbor method to determine the smallest *m*, and ensured that *m* and τ were robust based on prescriptions suggested by methods of Abarbanel *et al*.^59^. The basic idea is that by decreasing the dimension an increasing amount of phase space points will be projected into the neighborhood of any phase space point, even if they are not real neighbors. We used average mutual information to determine τ. The embedding dimension and lag values were calculated for all the time series and one value of each was chosen for the recurrent maps. The determined *m* = 6 and average τ = 25, excluding any outliers were used.

### Calculation of recurrence measures

The phase space reconstruction of CoM and platform was used in order to estimate the characteristic of the dynamical system based on the specified methods^60^. The resultant two-dimensional cross recurrence plot showed the recurrence of the two time-series in the reconstructed state space. The recurrence of the two time-series is then reduced to a two-dimensional binary plot where each column represents one point in the evolution of one time-series and each row represents one point in the evolution of the other time-series. An *isolated dot* was placed on the plot at any point where the two time-series are recurrent. Thereafter, we calculated the following cRQA features of the recurrence plot that inform about the different dynamics of the system:

#### Mean diagonal lines

Average amount of time the two signals are moving together in state space, longer mean *diagonal lines* reflects that the CoM and platform movements move together for a longer time on average, hence a stronger synchronization.

1 $${R}_{i+k,j+k}=1$$

for *k* = 1, …. *l*, where *l* is the length of the *diagonal* line.

#### Mean vertical lines

If one system “pauses” in state-space while the other one moves forward, a vertical line will appear. In this context of postural balance, it indicates an intermittent type of control, where magnitude of CoM and platform would have different movements, i.e. it would be able to delineate periodic-chaotic processes from deterministic processes.

2 $${R}_{i,j+k}=1$$

for *k* = 1, …. *v*, where *v* is the length of the *vertical* line.

#### Entropy of *diagonal* and *vertical* lines

This is the Shannon entropy of the frequency distribution of line lengths (*diagonal* or *vertical*) that reflects the complexity of the deterministic structure. Calibrated in bits/bin, the higher the entropy the more complex the dynamics, e.g., for uncorrelated oscillations the value of entropy is rather small, indicating its low complexity. In this context, tightly synchronous oscillators should have longer entropy *diagonal lines*, and periodic-chaotic states should reveal shorter entropy *vertical lines*.

3 $$ENT=-\,{\sum }_{l=\,{d}_{{\min }}}^{N}p(l)\mathrm{ln}\,p(l),{\rm{where}}\,p(l)={H}_{D}(l)/{\sum }_{l=\,{d}_{{\min }}}^{N}{H}_{D}(l))$$

where, ${H}_{D}(l)$ is the histogram of the *diagonal lines*, *N* is the number of considered states.

#### Radius (R)

This Euclidian radius was used as a measure to calculate the tolerance in the state space. We initialized the radius as 0.01 for the starting point and adjusted until the rate of recurrence reached 2.5%. To reveal the proper dynamics, our strategy was to calculate the recurrence for several increasing values of *radius*. In other words, if the system is very recurrent then it would not take a very large radius to find the 2.5% recurrence, whereas if the system is not as recurrent then the radius has to be larger, i.e. we accepted *isolated dots* farther apart to find recurrence. Here, an ideal recurrent state is 0, while higher non-recurrent states will have a proportionate higher value.

#### Determinism

It is defined as the fraction of recurrence points that forms diagonal lines. Systems possessing deterministic (rule obeying) dynamics are characterized by the *diagonal lines* and can be interpreted as the predictability of the system more so for periodic behaviors than chaotic process.

4 $$DET={\sum }_{l=\,{d}_{\min }}^{N}l\,{H}_{D}(l)/\sum _{i,j=\,1}^{N}{R}_{i,j}$$

where, ${d}_{min}=2$ and *N* = corresponds to the data points for each platform frequency.

#### Laminarity

From vertical lines, additional information regarding the percentage of recurrent points in *vertical lines* can be obtained. Since *laminarity* quantifies the relative amount of vertical structuring over the entire recurrence map, it also represents the frequency of *laminar states* (intermittency) within the system. *Laminarity* will decrease if the recurrent map contains recurrent points that are more isolated than in *vertical* or *diagonal* structures. Alternatively, this indicates the trapping time that reflects how long the phase space trajectories are trapped in the system.

5 $$\begin{array}{c}\,\,\,\,\,\,LAM=\sum _{l={v}_{min}}^{N}l\,{H}_{V}(l)/\sum _{i,j=\,1}^{N}{R}_{i,j}\\ {\rm{where}}\,{H}_{v}(l)=\sum _{i,j=\,1}^{N}(1-{R}_{i,j-1})(1-{R}_{i,j+l}){\prod }_{k=0}^{l-1}{R}_{i,j+k}\end{array}$$

### Statistical Comparisons

There was no statistical difference between trials within a condition or between visits. Therefore, the resultant data variables were averaged across the 12 trials for each frequency. Separate one-way repeated measures ANOVAs were used to test for the effect of frequency on mean line length and *entropy* values for *diagonal* and *vertical* lines, *radius*, %*determinism*, and %*laminarity*. For dependent variable post-hoc comparisons, we applied Tukey’s HSD procedure.

### Data availability

Available at journal’s repository.
